# Prescription medicine sharing: exploring patients’ beliefs and experiences

**DOI:** 10.1186/s40545-016-0075-5

**Published:** 2016-09-09

**Authors:** Kebede Beyene, Trudi Aspden, Janie Sheridan

**Affiliations:** The School of Pharmacy, Faculty of Medical and Health Sciences, the University of Auckland, Private Bag 92019, Auckland, 1142 New Zealand

**Keywords:** Medicine sharing, Medicine borrowing, Medicine lending, Non-recreational sharing, Auckland

## Abstract

**Background:**

Prescription medicine sharing has been defined as the lending of medicines (giving prescription medicines to someone else) or borrowing of medicines (being given and using a medicine prescribed for another person). This qualitative study explored the views of patients, to elicit information regarding factors influencing medicine sharing behaviours, their experiences of the consequences of prescription medicine sharing, and their risk assessment strategies when deciding to share.

**Methods:**

One-on-one, face-to-face, semi-structured interviews were carried out in Auckland, New Zealand between September 2013 and August 2014 with 17 patients, purposively sampled to provide information from different socio-demographic backgrounds. The interviews were audio recorded, transcribed verbatim and analysed using a general inductive approach. The study received ethical approval, and all interviewees provided written informed consent.

**Results:**

Findings were captured within five overarching themes: types of shared medicines; perceived benefits of sharing medicines; negative experiences of sharing; factors influencing sharing behaviours; and risk assessment strategies. Participants reported that sharing helped them to avoid treatment costs and the inconvenience associated with medical visits such as booking appointments. Conversely, unanticipated side effects, allergies, and taking inappropriate medicines were the main adverse consequences of sharing. Altruism, limited access to medicines/health services, sociocultural factors, and having unused prescription medicines were factors influencing sharing behaviours. Participants reported assessing the safety of sharing a medicine primarily based on symptom matching, past illness experiences, and knowledge about the medicines.

**Conclusions:**

This study enriches previous survey findings, by providing insight into patients' reasons for medicines sharing. Healthcare providers should consider asking their patients about any medicines they have shared and their future sharing intentions, in order to use the opportunity for discussing safer sharing practices, without promoting the behaviour. The findings are helpful for informing the development of potential interventions and targeted educational messages about safe medicine use for patients.

**Electronic supplementary material:**

The online version of this article (doi:10.1186/s40545-016-0075-5) contains supplementary material, which is available to authorized users.

## Background

Prescription medicine sharing has been defined as the lending of medicines (giving prescription medicines to someone else) or borrowing of medicines (being given and using a medicine prescribed for another person) [1]. Medicine sharing has two distinct types; these are recreational and non-recreational sharing. While recreational sharing can be defined as the sharing of abusable prescription medicines “to get high” or feel good, non-recreational sharing is the sharing of any prescription medicine for its intended therapeutic benefits. Although most forms of sharing are non-recreational in nature, [2] recreational sharing has received most of the research focus for the previous four decades [3–5]. Limited research evidence is available around non-recreational sharing; therefore, this study aimed to fill this gap.

While non-recreational sharing is not a new phenomenon, it has received increasing attention from researchers, due to its potential medical and public health implications [1, 6–16]. Some of these implications are unmonitored adverse drug events, [6] drug resistance, [7] complications in clinical diagnosis, [6] and delay in care seeking [17]. Although borrowers are at higher risk than lenders, of unexpected side effects or allergies from shared medicines, [9] lenders could also deplete their own medicine supply and incur a risk of therapeutic failure. On the other hand, medicine borrowing may be beneficial in some instances, for example, in an emergency situation. For those who do not have easy access to health services or are dissatisfied with healthcare systems, sharing provides an alternative method for accessing prescription medicines [18–20]. Furthermore, in modern societies, medicines are often part of an individual’s everyday life and might be shared to restore ill health or to facilitate social interactions [19].

A range of medicines has been reported to be shared [1, 8–11, 13, 20–26]; pain medications, allergy medications, and antibiotics were chief among them [1, 10, 23]. The sharing of asthma inhalers, [20] diabetes medications, [11] antihypertensives, [8, 10] corticosteroids, [21] acne medications, [21, 23] and antiretrovirals [25, 26] has also been reported to be common. Friends and family members were the main sources of shared medicines [1, 8, 23, 27]. Though medicine sharing is frequently reported amongst adults and adolescents, [9, 23] one study reported that the parental use of a child’s prescribed medicine for another child also occurs [10]. While this form of sharing could help to alleviate a child’s illness, administration of medicines not meant for a specific child could result in using inappropriate doses, hospital admission, or death [24].

Although there is no accepted, gold standard method for assessing medicine sharing, 5–66 % prevalence rates of sharing have been documented across studies [1, 8, 9, 11, 13–15, 21–23, 27, 28]. Females have been reported to be more likely than males to lend prescription medicines [1, 21, 23, 27]. In a representative national sample of 26,566 American adults, age was found to have more influence than gender on medicine lending or borrowing behaviour, with the prevalence of lending or borrowing behaviour increasing as age decreased [23]. Ethnicity and/or race appear to have less influence on medicine lending behaviour [29]. Lower income level, [27] unemployment, [10] larger household size, [10, 27] and having a chronic disease [21] have been found to be positive predictors of sharing behaviours, across studies. On the other hand, one study found that having health insurance, and a primary care provider who frequently asked about medicine usage were negatively associated with the borrowing behaviour of urban medical centre clients in New York [8].

Several factors have been reported to be reasons for medicine sharing behaviours. These include running out of previously prescribed medicine, [1, 10, 11, 23] inability to afford a medical visit or prescription charges, [8, 11, 13, 27, 30] emergency situations, [1] or convenience [8]. Being prescribed the same medicine or having the same health problem as the other person could also create opportunity for sharing medicines [1, 22]. Although several reasons contributing to medicine sharing were revealed by the above studies, many of these studies listed predetermined factors and asked participants to choose among them, [1, 9, 11, 14, 23, 27] and this process might limit the identification of a comprehensive range of motives for sharing from the patient perspective. Overall, little is known about the psychosocial, behavioural and cultural factors that influence medicine sharing. It is also unclear how patients assess the risk of sharing medicines and if, and how they assess risk in their decision making to lend/borrow medicines.

Further, previous research around sharing has tended to take a medical perspective and largely focussed on negative consequences of sharing behaviours [1, 6, 7, 17]. This approach may not provide a complete picture of sharing. People’s decisions to share medicines might not differ from their decision to share other commodities, [31] and understanding the ‘social context’ of medicines could provide further insights into why people share medicines.

The majority of the evidence around adults’ medicine sharing practices has been from cross-sectional surveys; limited qualitative studies are available describing non-recreational medicine sharing behaviours among adults [12]. A recent, extensive literature search by the research team has revealed only two qualitative studies that were specifically designed to assess medicine sharing behaviours [11, 31]. One of these studies explored older adults’ sharing behaviours, [11] and the other was designed to determine people’s willingness to share medicines in comparison with other sharable commodities and, as such did not provide details about sharing behaviours [31]. Our study has addressed this gap in the literature. The overall research process was guided by the central research questions: “Why do adults lend or borrow prescription medicines" and "How do they decide whether or not to do so?” These questions seek to understand sharing behaviours from medical and socio-cultural perspectives. The aims of the study were: (1) to understand adults’ experiences of the consequences of prescription medicine sharing; (2) to identify factors influencing medicine sharing behaviours; and (3) to understand how patients decide if prescription medicines are safe to be shared. This study has provided new information regarding the types of shared medicines, factors contributing to and potential consequences of sharing and patients’ medicine sharing risk assessment strategies. Therefore, the information may help healthcare providers to identify and address problems related to medicine sharing. The findings can also be used by patient support groups, medicine regulatory agencies, and other local and international organisations involved in promoting the safe use of medicines.

## Methods

### Design and sampling

A qualitative approach was adopted for this study. One-on-one, face-to-face, semi-structured interviews were conducted to explore adults’ beliefs and experiences about prescription medicine sharing practices. In this type of research it is usual to recruit up to 30 participants or collect data until data saturation is reached, whichever comes first [32]. Participants were sampled purposively based on their age, gender, income, and employment status in order to gather a wide range of information from different socio-demographic backgrounds. English-speaking adults aged 18 years or older with experience or interest in medicine sharing were eligible to participate. Health-care professionals were excluded from participation. The interpretation of findings was guided by a ‘harm reduction’ philosophy focusing on minimising the risks/harms of sharing without necessarily considering it essential to eliminate sharing practices.

### Study setting and recruitment

The study took place in Auckland, the largest city in New Zealand with a population of 1.4 million [http://www.statistics.govt.nz/Census/2013-census/profile-and-summary-reports/quickstats-about-a-place.aspx?request_value=13170&tabname=]. Participants were invited through local patient support group newsletter advertisements, pamphlet advertisements in a public library and a community pharmacy, and via email advertisements on university email lists. When participants approached the researchers, their eligibility was checked by the first author (K.B.) using a checklist developed for this purpose.

### Data collection

To ensure consistency, all interviews were conducted by K.B. K.B. is a pharmacist, but not registered to practice in New Zealand, and this was made known to participants. An interview guide (see Additional file 1) was used to ensure broadly similar topics were covered with each participant whilst allowing them to introduce and expand upon individual experiences. The selection of topics was guided by the research team experience and a review of available literature [1, 8, 13, 16, 17, 23, 27]. The research proposal was also presented at School of Pharmacy, the University of Auckland, departmental seminars and feedback received from colleagues was used to refine the topics. The interview guide was piloted with two participants and the results discussed among the research team to further refine the guide. Pilot data were excluded from final analysis. The main topics explored in the final guide were: the types of medicines shared by participants, participants’ beliefs and experiences about benefits and adverse consequences of medicine sharing, circumstances that lead to sharing medicines, participants’ views regarding ways to minimise the potential harms of medicine sharing. After conducting the first four interviews, previously unforeseen topics were added to the interview guide. Participants also completed a short questionnaire describing their socio-demographic characteristics and medicine-taking habits.

Participants were interviewed at a time and location of their choice. With the exception of one participant (who preferred to be interviewed at home), all interviews were conducted at the University of Auckland. To ensure participants understood the purpose of the study, all participants were provided with a general definition of medicine sharing, and were instructed to focus on non-recreational prescription medicine sharing. With participants’ consent, all interviews were audio recorded. At the end of each interview, the participant was given a document describing the potential risks of medicine sharing. Interviewing continued until data saturation was reached, which was considered to have occurred after 17 interviews (as no new themes were identified from the last three interviews). The mean duration of the interviews was 47 min (range: 30–72 min), and they were conducted between September 2013 and August 2014.

### Data analysis

After each interview, the interviewer made field notes and this information was used to describe how each interview was conducted and to note issues or comments not sufficiently captured by audio recordings. This information aided interpretation of data. Interviews were transcribed verbatim by K.B. (*n* = 6) or an independent professional transcriber (*n* = 11), and the transcripts were checked against the audio recordings by K.B. All participants were offered an opportunity to edit their interview transcripts; however, with the exception of one participant, they did not read and edit them. To ensure anonymity, names, place of work, or any other identifying information was removed, and a unique code number was assigned to each transcript. The data were analysed using the general inductive approach (GIA) [33]. GIA is a thematic analysis approach with both deductive and inductive features. In GIA, while the general (or overarching) themes are derived from the research objectives (deductive feature), more specific categories/themes arise from the data (inductive feature) [33]. NVivo 10 software (QSR International) was used to support the analysis process. Before data coding began, the audio recordings, field notes, and the interview transcripts were repeatedly revisited by K.B. to identify salient topics in relation to the research objectives. The preliminary findings were also discussed amongst all authors at several meetings held during the course of the project. Initially, the authors met to agree on the coding procedure and a coding framework was developed to guide the process. Then, two of the authors (K.B. and T.A.) independently coded four, randomly selected interview transcripts, and then all authors met to resolve any coding discrepancies. The main discrepancy identified was an overlap between themes; for example, sharing medicines because of inability to pay for a medical visit was coded initially both under ‘cost’ and ‘access’ codes, but later a decision was made to code these data as ‘cost.’ Discrepancies and overlaps were remedied through mutual consensus among the research team. Afterwards, the codes from both coders were merged to create an initial codebook. Using the initial codebook, K.B. coded the remaining transcripts. The initial codebook was added to and adapted as the coding process progressed to incorporate emerging codes. Instead of line-by-line coding, the coder coded only sections of data meaningful to the research aims. Overall, two levels of coding were employed. The initial coding phase was used to reduce large tracts of unorganised data into a more manageable form based on the general research topic. The second phase involved more focused coding and was used to identify themes, patterns and relationships emerging across the data. The initial and second phase codes and their text segments were systematically organised to examine for any similarities or differences in order to draw conclusions. At this point, patterns and relationships started to emerge within and among major themes and sub-themes (see Table 1 for details). Information from each interview was compared with other interviews to elicit variation and to identify similarities and explain outlying data or negative cases. Mind maps and diagrams (drawn using Post-It notes and poster paper) were used to visualise the relationship between themes and sub-themes. After completion of coding, the data within each code were re-read and this process helped to identify and accurately re-code miscoded data into appropriate categories. The authors then met to discuss the main findings and to explore alternative interpretations of the findings. Overall, five overarching themes were identified. These were: types of shared medicines, perceived benefits of sharing, negative experiences from shared medicines, factors influencing sharing medicines, and risk assessment strategies.

**Table 1:** Overview of codes, sub-themes and overarching themes

Codes (specific categories)	Code description	Sub-theme	Overarching theme
Pain, asthma and sleep medications, antibiotics, and so forth.	Classes of medicines shared by participants	NA	Types of shared medicines
Avoid doctor visit	Sharing avoids the need to visit a doctor	Saves time and money	Perceived benefits of sharing medicines
Avoid hidden costs	Sharing avoid taking time off work or avoid inconveniencing work – avoids hidden cost		
To avoid waste	To avoid buying a packet when only need one dose or might not work		
Convenience	Sharing is more convenient, the medicines are readily available, no need to visit a GP		
Emergency	Sharing when someone is in great need of the medication or during emergency		
To try the medicine	To see if the medicine works before obtaining a personal supply		
Misplacing medicines	Misplacing own medicines and temporarily sharing other’s medicines		
Common minor condition	Sharing medicines when the patient perceives that the medical condition is minor		
Caring relationship	Sharing is a means of supporting each other during illness – caring relationship	Social support	
Inappropriate dose/wrong medicines	Person takes inappropriate doses or wrong medicines	Unsafe and ineffective treatment	Negative experience from shared medicines
Adverse drug events	Sharing might result in unanticipated side effects, drug interactions, allergy or contraindications		
Expired medicines might be shared	Expired medicines might be shared		
Risk of killing/harming	Sharing may have a risk of killing or harming a person		
Topical medicines are weaker than orally ingested medicines	Sharing topical medicines is not as risky as sharing pills – can be removed by washing		
Loss of medication instruction	Lack of information – e.g. borrower does not have information on risks, adverse outcomes, etc.		
Medical condition get worse	Sharing complicates simple medical conditions by delaying diagnosis and treatment		
Misdiagnosis	Sharing based on misdiagnosis could be dangerous		
Unhygienic	Sharing medicines (e.g., inhalers) is unhygienic	Public health risk	
Antimicrobial resistance	Sharing might increase drug resistance		
Spread infection	Sharing creams/ointments might spread the disease – cross infection		
Affects social relationship	Sharing addictive medicines may affect one's personal relationship with others	Risk of drug dependence	
Dependence	Sharing might result in drug dependence		
To help a friend or family	Sharing to help out others or to make someone feel better – caring relationship	Altruism	Factors influencing medicine sharing
Ran out	Ran out of previously prescribed medicines		
Cost	Sharing saves doctor's fee, prescription charges, or cost of unsubsidised medicines	Limited access to medicines/health services	
Access	Sharing when difficult to access medicines – for example prescription restriction and when pharmacy or doctors are inaccessible or where there is no nearby health facility		
Waiting times	Sharing medicines to avoid long appointment or waiting time at GP surgeries		
*‘After hours’*	Sharing for pain occurring late at night or over the weekend – when a regular GP is not accessible		
Traveling	Sharing medicine during family trip, holiday trips or when traveling overseas		
Forgetfulness	Someone forgets to carry around their own medicines		
Leftover medicines	Having leftovers/unused medicines creates opportunity for sharing	Leftover medicines	
Lack of information about safe disposal	Not knowing what to do with leftover or unused medicines		
HCPs not mentioning not to share	When patients do not receive information from health care providers about the risk of sharing		
Cultural influence	Cultural beliefs, family values and customs may influence medicine sharing	Sociocultural factors	
Embarrassment	Embarrassment about seeing a doctor or embarrassed to carry around own medicines		
Ads/Internet	TV ads or the Internet encourages self-diagnosis and sharing medicines		
Familiarity with the medical condition or the medicine	Familiarity with the medical condition or the medicine– facilitates sharing	Experience of, and knowledge about illness and its treatment	Risk assessment strategy
Complex medical condition	If the condition is complex – deterrent		
Uniqueness of medication for the person	Medicines meant for a specific condition are less likely to be shared		
Unaware of risk	Unaware of risk of sharing – facilitator		
Perception of efficacy	Assuming if the medicine worked for the lender it will do the same for the borrower		
Perceived danger of medicine	Concern about side effects – deterrent		
Borrower’s responsibility	Borrower decides and accepts responsibility for consequences	Borrower’s responsibility	
Same symptoms	Having the same symptoms facilitates sharing	Symptoms matching	
Same medicines	Attitude that taking similar medication as the other person might not have a negative effect on one’s health		

*NA* not applicable

## Results

### Participant characteristics

The sample comprised 12 females and five males, with a mean age of 41.2 years (Range: 23–69 years) and the majority of participants (n = 10) self-identified as New Zealand European (see Table 2 for detailed description of participants’ characteristics). Twelve participants were recruited through email advertisements to the University of Auckland community, and two each through a public library and a patient support group. One participant was recruited from a community pharmacy.

### Types of shared medicines

A range of medicines was reported to be shared (see Table 3). Although most of the sharing practice described was for medical purposes, sharing for non-medical purposes was also reported, for example, for cosmetic use (e.g., Botox^®^), to relax or to “get high” (e.g., strong pain medications), and for dietary supplementation (e.g., glucosamine). Participants also reported sharing homeopathic preparations which were sometimes recommended by their GP.

**Table 2:** Participant characteristics

Characteristics	Number (*N* = 17)
Gender	
Male	5
Female	12
Age in years^a^ (Mean age = 41.2 years, Range: 23–69 years)	
20 - 30	6
31 - 40	2
41 - 50	3
51 - 60	2
60+	2
Ethnicity	
New Zealand European	10
Chinese	4
Others (Indian, Māori, Brazilian)	3
Highest level of education attended	
Tertiary education (college and above)	15
Secondary school	2
Working status	
Working full time	10
Working part time	1
Retired	2
Student	3
Unwaged	1
Monthly income (in NZD)^a^	
<1000	2
1001 – 2000	7
2001 – 4000	2
4001 – 6000	2
6000+	2
Have you been taking any prescription medicines in the past one year?	
Yes	16
No	1

^a^Data were not obtained from two participants

**Table 3:** Medicines shared by participants

Allergy medications (e.g., e.g., Zetop^®^, EpiPen^®^, hay fever medications)
Antibiotics (e.g., amoxicillin, Augmentin^®^)
Antidiarrhoeal medications
Antiemetic medications
Antihypertensives
Anti-inflammatory medications (e.g., diclofenac, naproxen)
Asthma inhalers (e.g., Ventolin^®^, Symbicort^®^ inhaler)
Botox^®^ (cosmetic use)
Cardiovascular medications (e.g., Cartia^®^, aspirin)
Cholesterol medications (e.g., simvastatin)
Constipation relief medications (e.g., VitoLax^®^)
Diabetes medications (e.g., metformin, glipizide)
Dietary supplements (e.g., glucosamine tablets)
Gastric/duodenal ulcer medications (e.g., omeprazole, Losec^®^)
Homeopathic medications
Hypnotics (e.g. nitrazepam, Valium^®^, melatonin)
Migraine medications (e.g., sumatriptan)
Muscle relaxants
Nasal spray to treat snoring
Nitrous oxide canister
Oral contraceptive pills
Pain medications (e.g. tramadol, morphine, codeine, pethidine)
Psoriasis medications
Topical antifungal/corticosteroids (e.g., Micreme H^®^, Betnovate^®^, eczema medications)

### Perceived benefits of sharing medicines

Participants reported a range of benefits and these were categorised into two sub-themes.

#### Saves time and money

Participants stated that medicine sharing helped them to avoid doctors’ fees and prescription charges, and reduced the burden associated with medical visits such as booking appointments, arranging transport, and the need to visit a pharmacy afterwards. Participants stated that visiting a doctor often requires taking time off work and can be costly in terms of income loss or the inconvenience it can create. Participants also reported sharing prescription medicines for conditions that require immediate treatment because of long waiting times at busy surgeries. Sharing also provided participants with easy access to medicines particularly when they considered themselves not sick enough to visit a doctor or when they only required a few doses of the medicine.

For those who were concerned about finances, sharing provided an opportunity to try the medicine before making a decision to pay for a doctor’s appointment. When the shared medicine was found to be effective, some participants visited their doctor to inform them of their positive experience and obtain their own prescription.

*I have psoriasis and I have some friends who have psoriasis. So if they said to me, “Oh my doctor gave me this [cream], and it was great.” I might say, “Oh do you think I could try that a little bit?” I could see myself doing that. Maybe I’ll see if it seems to work for me and I’ll go to the doctor and get it myself.* (P07, 69-year-old female)

For some participants, medicine sharing was a way of preventing wastage of resources. Some considered discarding unused medicines to be wasteful, and was a reason they were willing to share their unused medicines, as long as they believed them to be of benefit to someone else. During many of the interviews participants asked the interviewer if there was any way to return or donate unused medicines. Also of note, was that participants considered paying for medical visits to be a waste of money, when only a few doses of medicine are required.

Sometimes medicines were lost or misplaced and participants borrowed to avoid the inconvenience of getting a replacement prescription. Sharing also allowed some participants to avoid the fear of the consequence of missing a few doses of the medicines they were relying on (e.g. diabetes medications or asthma inhalers).

#### Sharing as a form of social support

Apart from cost reduction, medicine sharing had a positive impact on social interactions. During sharing, participants disclosed the nature of their illness to the other person, and this process helped them to share illness experiences and to sustain and nurture a social relationship. A university student described her boyfriend’s positive experience as follows:

*My boyfriend got a nasal spray* [for snoring] *from his friend, that really helped, that really worked, even brought them closer, because that’s a very special experience.* (P08, 28-year-old female)

### Negative experience from shared medicines

Participants described various risks and harms of medicine sharing and these were classified into three sub-themes. While some participants revealed their own negative experiences from shared medicines, others provided hypothetical scenarios to explain the potential risks of sharing.

#### Unsafe and ineffective treatment

In an effort to help friends or relatives, participants engaged in “diagnosing” and “prescribing” which resulted in sharing their medicines with others. However, in so doing, some of the participants ended up giving inappropriate medicines to the other person. According to participants, it is often difficult for an untrained person to make the correct judgment regarding the dosage, potential contraindications, and side effects as this often requires technical knowledge about medicines. Sharing can have life threatening consequences; one of the participants recounted the negative consequences occurring when her mother borrowed a medicine.

*My mother, she was having pain. She had spondylitis so she was having severe pain and she was talking to her friend who is her next-door neighbour. So she said, “Oh you know what, I have this medicine and it worked fantastic for me” and she gave my mum, and she was given a CNS acting drug, she was given* [sic] *to take three tablets a day. My mum took the same dose and she went into coma that night*. (P10, 30-year-old female)

A few incidences of medicine sharing had resulted in allergic reactions. Most participants believed that the decision to share medicines often relies on symptom matching alone and that allergy histories might not be properly assessed. One participant described her negative experience as follows:

*A long time ago my mum and I were in [country], we were living in a small town, we shared medicine once, after my mum took the medicine her eyes became swollen and she stopped the drug, and she went to the GP, and the GP actually told her that she is allergic to that kind of drug.* (P08, 28-year-old female)

#### Public health risk

Participants had different opinions regarding the risk of antibiotic sharing. Some of them had fewer concerns about sharing antibiotics particularly for common conditions; others were worried about treatment failure and antimicrobial resistance due to sharing and incomplete courses of antibiotics. Depleting one’s own supply and ineffective treatment were other risks mentioned by those who had concerns about antibiotic sharing.

Sharing some forms of medicines (e.g. eye drops, asthma inhalers, creams or ointments) was considered by participants to be unhygienic and a way of spreading infections. Apart from the spread of infections, it appeared that participants had fewer concerns about the effects of some of these medicines. For instance, many participants were less concerned about the possible side effects or toxicity from shared creams or ointments, compared to pills. On the other hand, one participant warned that the likelihood of exacerbating skin conditions when using inappropriate creams could be high, and she explained the common misconceptions towards topical preparations amongst the public.

*We think it’s safer because we don’t take it in and that’s less chance of getting poisoned by it or something. It’s probably not, that’s what people think. If you eat it inside then people take more precautions. Whereas if it’s topical like something on the skin, like if it’s a cut or a burn or something then they just think it’s less important than something that’s happening inside.* (P16, 29-year-old female)

#### Risk of drug dependence

Participants also discussed about the impact of medicine sharing on drug dependence. Participants stated that although sharing by itself might not necessarily result in drug dependence, some medicines (e.g. strong pain medications) might be shared for thrill-seeking. Participants believed that this form of sharing could have a negative impact on social relationships. A participant who had been prescribed these medicines described strategies he uses to minimise the chance of being a source of abusable medicines, such as keeping social distance or concealing information from potential abusers.

*If they [drug abusers] knew what I had [strong pain killers] they would be knocking at my door, “bro can I have a few of these, few of that?” So that’s why I tell them nothing because I know what they use them for.* (P11, 50-year-old male)

### Factors influencing medicine sharing

Participants reported many factors that could facilitate prescription medicine sharing practices; these were classified into four sub-themes.

#### Altruism

A desire to help others (i.e. altruism) was often the main motivator for those who reported sharing medicines. They shared their medicines for what they perceived to be a good reason, to help their loved ones when they were suffering from an illness. Participants’ past illness experience could further encourage altruistic behaviour. This was described by one of them.

*Just someone here at work had a terrible migraine and the person did not have access to medication, you know, and it’s something that I suffer from migraine and I know how horrible it is so I just said look I have sumatriptan here, if you want I can give you.* (P12, 41-year-old male)

Although medicines were more often reported to be shared with family members, close friends, neighbours, or work mates, when it came to helping people in urgent need of medicine, social distance was less important.

*Yes, one time actually, my boyfriend has asthma and I had his inhaler in my bag because I always carry around for him, and I was out in the pub and a guy had really bad asthma and his friend was running around and saying “Inhaler, inhaler”, so I gave him, so yeah, I did do that, I am happy to do it, it was not like an emergency, but he definitely needed the inhaler.* (P09, 25-year-old, female)

Sometimes the lender used the sharing instance as an opportunity to establish a friendship with the borrower, although the main motivator, in doing so, was often altruism.

*I get anxious so I take omeprazole or Losec*^*®*^*. … And if somebody told me that their stomach was hurting and they needed one of those, I would do that* [share]*, sure. I’m trying to be friendly and a bit of a socialist, you know (laughs), but I don’t do it to save money.* (P07, 69-year-old female)

#### Limited access to medicines/health services

The cost of medical visits hindered participants with limited resources from seeking medical care and being prescribed their own medicines. Some of them revealed that their health insurance was limited and did not cover all their medical needs, for example, students and retired participants. Limited opening hours of local pharmacies and surgeries also influenced participants’ sharing behaviours. For instance, participants reported sharing medicines for pain occurring late at night or at the weekend when local health facilities were not accessible. A university student associated her medicine sharing behaviour with the campus clinic opening hours as follows;

*My health insurance is very limited, for example, it’s only free if I go to the campus clinic, and the campus clinic you need to book and their opening hours is very short, I think they are closed at 3:00 PM or 4:00 PM. So it will be easier if I just get the medicine from someone I know and solve my problem.* (P08, 28-year-old female)

Participants also reported sharing medicines while travelling when they could not access their regular doctor within a reasonable time.

#### Sociocultural factors

Social, cultural, and interpersonal interactions influencing participants’ sharing behaviours were captured by this theme.

Although not nationally representative, participants were drawn from different communities, and it was noted that for some participants (e.g. those recently migrating to New Zealand) visiting a doctor and getting their own medicines was not an easy experience, partly because of cultural differences (e.g. communication barriers or differences in the health systems). Those participants considered medicine sharing to be a means of accessing prescription medicines without having to see a GP. It was also revealed that in some cultures sharing medicine and other commodities is a way of providing social support for others. One participant had lived in New Zealand for a number of years. He described the difference in perspectives about sharing medicines and the role of medicines, between people from his homeland and New Zealanders, as follows;

*In New Zealand it’s not culturally acceptable, you don’t share someone else’s drink whereas back home it is quite common so I guess that cultural difference may come into play.… [people from his homeland] are much more interventionist they will medicate people much more commonly than in New Zealand. So back home, I think there’s much more medication sharing than in New Zealand.* (P12, 41-year-old male)

Embarrassment was reported as a major reason why some patients do not visit their GP to obtain their own prescriptions. Participants also believed that younger patients might be embarrassed to carry around medicines they rely on and need to take in front of others (e.g., asthma inhalers) because doing so might be considered, by peers to be an indication of weakness.

Participants believed that advertising medicines on television might encourage people to self-diagnose and borrow medicines. It was noted by participants that when a prescription medicine is advertised on television it may lead the public into believing that the advertised medicine is safe to be shared, and might change their attitude towards the risks associated with sharing it. Younger participants appeared to be more influenced to share medicines by patient fora and various drug information sources on the Internet. One of the younger participants reported surfing the Internet to help her decide whether to take the medicine she had been offered by a friend.

*I remember once I had a cat bite, the cat got some fungus infection and I got the infection on my skin as well. I talked to some friends who also had a cat and I asked whether they had this kind of problem as well, and yeah she said she had once a cat bite and she still have the ointment. So I Googled the drug and I thought that it suits my condition and I borrowed from her, and I just used it until the infection was gone, until I recovered.* (P08, 28-year-old female)

#### Having leftover/unused prescription medicines

Participants were unsure what to do with unused medicines and many of them mentioned passing their unused medicines on to others. Most of the participants revealed that they received little information from healthcare providers about the safe disposal of leftover medicines. Some participants criticised their doctors for failing to understand their actual need and for supplying them surplus pills. A retired woman with back pain was among those who were unhappy with their doctor’s prescribing behaviour.

*Sometimes I have a really bad back problem and so for a little while I went to a back person* [doctor] *and I think they’re very negligent. He gave me a million tramadol, you know, it was like so much and they just give you these giant prescriptions and so I don’t think I would share that tramadol. To me I would put that into a more serious; it’s not like an aspirin.* (P07, 69-years-old female)

Although the above participant did not share her tramadol this was not the case for all participants. Some of them reported sharing their spare medicines (e.g. antibiotics), and they believed that having an oversupply of medicines motivated them to do so.

### Risk assessment strategies

Participants reported different strategies that helped them to assess whether it was safe for them to share medicines and these were classified into three sub-themes.

#### Experience of, and knowledge about illness and its treatment

Their own experience with the medicine also helped participants to assess risks of sharing. If participants had been taking the medicine and if they knew what it was for and were confident that it could help the other person, they were more likely to share the medicine. On the other hand, if participants thought that their medicines were specific to their condition, if they were unfamiliar with the medicine or when they were not confident enough about the borrower’s medical condition they were less likely to share.

Knowledge about illness and its treatment could also play a significant role in decisionmaking. For example, those who were aware of the risk of misdiagnosis of bacterial infections, risk of allergy and/or antimicrobial resistance firmly opposed sharing oral antibiotics. However, most participants were unaware of the potential risks of topical antimicrobials and were willing to share them. Although the meaning of ‘complicated condition’ was different among participants, they were also generally uncomfortable with sharing medicines which are meant for conditions such as cancer, diabetes, heart problems, or hypertension.

Friends’ positive experiences with a medicine and obtaining the medicine from a trusted person (i.e., a person with good knowledge of medicine or who used it for long time) also had an influence on participants’ decisions to borrow medicines.

#### Borrower’s responsibility

Some participants were not concerned about assessing risks when lending medicines. They believed that they were not qualified to diagnose the borrower’s condition or to assess the risk of the medicine. Further, they stated that it is up to borrowers to decide if the medicine suits their condition and/or to check allergic reactions.

*I assumed that he didn’t have any sort of particular allergies or something towards the chemical but I think it’s not up to the person who’s offering the medicine to diagnose the other person and give him the right drugs. It’s just I have this, maybe you want to try it, maybe you don’t – I don’t really care.* (P14, 23-year-old female)

#### Symptoms matching

Symptoms matching was another strategy used by participants to assess the possible risks of sharing medicines. Participants believed that if someone had similar symptoms to the other person then the medicine would most probably work for him/her and be safe. Participants also frequently reported that they would be willing to try a medicine which worked for their friends with a similar condition to see if it would also work for their condition.

## Discussion

This research explored the positive and negative consequences of, and factors influencing non-recreational medicine sharing practices of adults. Furthermore, the study examined factors considered by patients when deciding to share a medicine. These findings advance our understanding of non-recreational medicine sharing and provide further insights into the social, cultural, economic, and therapeutic aspects of medicine sharing. Although the potential risks of medicine sharing are well known to researchers [21, 22, 27] and regulatory authorities, [http://www.fda.gov/RegulatoryInformation/Legislation/FederalFoodDrugandCosmeticActFDCAct/] the non-medical aspects of sharing have been largely unexplored. From the medical perspective, medicine sharing is often considered to be undesirable behaviour [6, 17]. However, patients often see their sharing practices as positive and the study indicates that they often have sound justifications for doing so, as described in our findings.

In line with other research, a wide range of medicines were reported to be shared [1, 8–11, 13, 14, 21–23]. A number of previously unreported medicines were also reported; these include melatonin, muscle relaxants, Botox^®^, homeopathic preparations, and migraine medications.

With the rising cost of health-care, it has been noted that patients with lower incomes may adopt different cost-coping strategies such as intentional non-adherence, increasing debt burden, or cutting back (or economising) on necessities such as clothes and food [34]. Our findings suggest that medicine sharing may be another coping strategy. This finding is also in line with earlier studies reporting financial hardship as a reason for medicine sharing and underutilisation of available health-care services [19, 20, 30]. Also of note, in some developing countries the prevalence rates of medicine sharing are high (55–66 %) [14, 28] when compared with reports from developed countries (5–27 %) [1, 8, 10, 11, 23].

For most participants, medicine sharing was not a major concern. Some participants did not perceive their medicine sharing to be risky. Instead, they weighed up the risks and benefits of sharing particular medicines primarily based on their previous illness experiences and symptoms matching. In most instances, participants did not disclose the medicines they have shared or their sharing intention to healthcare providers; one possible explanation for this could be a fear of upsetting their healthcare providers. Overall, participants described a multifaceted system for determining the safety of the medicines they had shared. It appears that medicine sharing practices described in this study sample often involved a thoughtful decision making process rather than irresponsible behaviour or ignorance. However, although sharing medicines had many benefits for participants, we are concerned by some forms of sharing. For instance, opioid painkiller effectiveness for non-cancer chronic pain is controversial, [35] and its continuous use may diminish pain threshold levels or lead to substance use disorders [36]. Antibiotic sharing practices might result in therapeutic failure and may contribute to the emergence of antimicrobial resistance [1].

Van der Geest et al., in their anthropological study of pharmaceuticals, pointed out that medicines can be exchanged between individuals to facilitate social interactions [37]. The authors further noted that medicines are representations of ideologies and lifestyle. Our study findings support this; we found that “prescribing” for others and medicine sharing were common practices, and these practices had a positive impact on participants’ social relationships. Many of the participants had been offered medicines by friends or relatives for free, and the sharing decision appeared to be influenced by altruistic reasons rather than the expectation of a reward. Therefore, any efforts to design interventions need to consider sharing behaviours within the context of wider social interactions.

Inconvenience, and embarrassment about seeing a doctor were other reasons why some participants shared medicines. This may be a coping strategy by patients in response to a healthcare system that does not address their needs and expectations. Language, cultural barriers and a lack of information about the healthcare system were reported to be contributing factors to poor access to health services and medicine sharing practices amongst immigrants in New Zealand [38] and elsewhere [39]. Similarly, immigrants in this study reported challenges they have faced in accessing medicines or health services (such as limited health insurance, an inability to pay doctors’ fees, and language barriers), particularly upon their arrival in New Zealand, and the contribution of those challenges towards medicine sharing practices. Providing more information for migrants on the healthcare system and using translators in healthcare facilities were suggested as strategies to reduce some of the problems mentioned [38].

This study also revealed that some participants initially try to self-medicate their problems with borrowed medicines before visiting their doctor. This behaviour might lead to a delay in clinical diagnosis and an increase in complications from simple conditions [6]. Moreover, if patients do not inform their prescribers they might be unaware of the patient’s sharing practices, and as a result they may prescribe medicines that should not be taken with the medicine the patient has borrowed [8]. Sharing unused/leftover medicines is also concerning. Some of these medicines might have passed their expiry date and/or their active ingredient might have deteriorated due to unfavourable storage conditions. Providing systems/pathways for unused/unwanted medicines to be safely disposed of (e.g. promoting collection of unused medicines by pharmacies) and prescribing medicine in quantities tailored to the actual need of the patient is more likely to reduce the amount of leftovers and associated sharing practices. Some of the adverse reactions reported by participants are potentially life threatening. However, no study to date has tracked the extent and severity of adverse drug reactions occurring as a result of shared medicines, and this is an important area for future research.

Overall, the findings indicate that some individuals are sharing 'high risk' medicines and this suggests the importance of incorporating questions assessing medicine sharing behaviours into prescribing, dispensing and medicine reconciliation activities. Available electronic medication records can assist in the process of identifying individuals sharing medicines. Posters and leaflets which show the potential risks of sharing medicines could be used to increase risk awareness. In addition, non-compliance with medication regimens is likely to increase the amount of leftover medicines, thus adherence counselling is crucial to reduce the opportunity for sharing.

This study is not without limitations and the findings should be interpreted in the light of these. Participants were not a representative sample of New Zealand adults, and thus their views may not be generalisable, although care was taken to recruit a sample from a broad range of backgrounds. This could potentially limit the transferability of findings. Those who could not speak English were excluded from the study. These individuals are more likely to have different cultural backgrounds and they might have unique concerns and expectations regarding their health and medicine sharing, particularly as language and communication barriers are more likely to increase medicine sharing.

As the professional qualification of the interviewer was made known to all participants, and it is possible that such information could have potentially influenced participants’ responses. For example, some participants might believe that sharing medicines is not condoned by some health professionals and may not have wanted to disclose the nature of some of their sharing practices to the interviewer. To minimise the potential for such social desirability bias, all interviewees were informed of the general purpose of the research and the measures taken to ensure confidentiality of information. They were further informed that there was no right or wrong answer to any of the questions. This appeared to put participants at ease. In some interviews the interviewer was challenged by participants to give his opinion on topics such as the possibility of sharing medicines in some instances and legal issues related to medicine sharing. However, such opinions were not supplied, except in instances where further explanation was sought relating to the intention of the interview questions. The study authors are pharmacists, and despite acknowledging both the benefits and harms of this practice, this could have influenced the study design and data interpretation. However, attention was paid to providing balanced perspectives. Lastly, the fear of punishment from a previous ‘bad’ experience about medicine sharing may have exclude some individuals from participation.

Limitations aside, this study revealed that medicine sharing is a behaviour more complex than some previous quantitative studies suggest. Accordingly, the findings have important implications for healthcare providers who regularly engage in patient consultations about the safe use of medicines and for future research on medicine use behaviours. A cross-sectional survey among larger cohorts is currently being conducted by the authors to examine the extent to which each identified factor influences patients' medicine sharing decisions, and the relationships between these factors.

Generally, as noted by Shoemaker and Ramalho de Oliveria, understanding medicine use behaviours requires carefully analysis of patients’ perspectives and should not rely only on health professionals’ perspectives [40]. Thus, taking into account how patients understand medicine sharing is critical in designing effective interventions to reduce harms from this practice.

## Conclusions

This study enriches previous survey findings, by providing insight into patients’ reasons for medicines sharing. For example, altruism, forgetfulness, cost of GP visits, illness denial and embarrassment, lack of awareness regarding the risk of sharing, which have received little attention in previous surveys, were identified as important factors influencing sharing behaviours. Health care providers need to be aware that the sharing of prescription medicines is not uncommon. The authors suggest that healthcare providers consider engaging patients in discussions which may provide further insights into factors influencing sharing behaviours for particular individuals. Overall, it might be impractical to stop people from sharing their prescribed medicines (and even undesirable under some circumstances), and minimising the potential risks/harms of sharing should, therefore, be a priority whilst also acknowledging the meaning such behaviours have for patients, and their reasons for sharing. Quantitative research is required to examine the extent to which the identified factors related to sharing influence patients’ decisions to share.

## Additional file

Additional file 1: Interview guide. (DOCX 17 kb)
