# Supplementary material for: Prescription medicine sharing: exploring patients’ beliefs and experiences
Source: J Pharm Policy Pract. 2016 Sep 9;9(1):23. doi: 10.1186/s40545-016-0075-5 (PMC5018191; doi:10.1186/s40545-016-0075-5)
Supplement: Additional file 1: — Interview guide. (DOCX 17 kb) [file 40545_2016_75_MOESM1_ESM.docx]

**Prescription medicine sharing: A qualitative exploration of patients’ beliefs and experiences**

Interview schedule

**Opening question**

1. What do you think prescription medicine sharing is?
2. What might be the benefits of lending or borrowing medicines?
3. What do you think are the disadvantages of lending or borrowing medicines?

**Lending**

1. Have you ever lent any prescription medicines to anyone? If No, why?

**If “YES”, the following questions will follow:**

1. Could you tell me in what circumstances you have lent your medicines?

Prompts: Did you lend the medicine to help out the person or for any other reason?

1. Who have you shared your medicines with?

Prompts: Have you ever lent to your family members, relatives, or close friends?

1. Which kind of medicines did you lend? And why?

Prompts: Where did you get these medicines from? Was the medicine a leftover?

1. Do you think sharing some types of prescription medicines is important? Tell me more about why you think this?
2. Can you think of any types of prescription medicines which are not safe to be lent?
3. Have you been influenced by TV/Radio/Internet Ads and commercials to lend medicines?
4. How did you decide whether the medicines were ok/safe to be given to that person?

Prompts:

1. Did you try to check if the medicine you have lent was suitable for the person? If yes, could you tell me more how you have checked if the medicine was suitable for the person?
2. Did you tell the person how to take the medicine you have lent? If yes, what did you tell him/her?
3. Do you think any possible harms or risks of lending prescription medicines?

Prompts: Based on the answer: Why do you think these are the major harms/risks? What do you think the possible ways to prevent these harms are?

**If the respondent answers either YES or NO to question #4, these questions will follow:**

- Could you tell me in what circumstances you would be willing to lend your medicines?

Prompts: Would you lend if someone from your family or friends ask you to borrow? Which of your prescription medicines you prefer to lend? And Why? Are you willing to give some of your prescription medicines for someone who is unable to afford for the medicines?

**Borrowing**

1. Have you ever borrowed a prescription medicine from someone else? If No, Why?

**If yes #5, the following questions will follow**

1. Which kind of medicines did you borrow? And why?
2. Was the medicine you have borrowed similar to those you had been taking?
3. Have you ever borrowed because of an inability to pay for medicines in the pharmacy or costs related to a GP visit? Was borrowing convenient for you compared to a GP visit to get prescriptions? Why?
4. Have you been influenced by TV/Radio/Internet Ads and commercials to borrow medicines?
5. Did the person who lent you the medicines give you written or verbal instructions and/or warnings for the medicines you have borrowed?

Prompts: What kind of instructions did you receive? Do you think the instructions you have been provided was sufficient to take the medicine safely?

1. Have you ever had a bad experience from taking borrowed medicines? If yes, please tell me more.

Prompts: Did your health condition get worse after taking the medicine you have borrowed? Have you ever had an allergic reaction from borrowed medicines? If yes, what did you do when you got the allergic reaction?

1. Did you visit your healthcare provider after borrowing medicines for your illness? What happened?

Prompts: Did you tell your doctor or pharmacist about the medicine you have borrowed during your visit for the same illness or during your other visits? If yes, can you tell me a bit more about that instance/case? If no, why? What advice/warning did you receive from your doctor or pharmacist when you inform them about your borrowing behaviour?

1. Do you think any possible harms or risks of taking someone else’s prescription medicines?

Prompts: Based on the answer: Why do you think these are the major harms/risks? How can these harms damage the borrower? What do you think the possible ways to prevent these harms are?

**For those who answer either YES or NO to question #5, these questions will follow:**

1. Would you be willing to borrow medicines from others?

Prompts: Under what circumstances? From whom? What type of medicines?

**Thank you very much for your participation in this interview**
